# Brainstem stimulation augments information integration in the cerebral cortex of desflurane-anesthetized rats

**DOI:** 10.3389/fnint.2014.00008

**Published:** 2014-02-24

**Authors:** Siveshigan Pillay, Jeannette Vizuete, Xiping Liu, Gabor Juhasz, Anthony G. Hudetz

**Affiliations:** ^1^Department of Anesthesiology, Medical College of WisconsinMilwaukee, WI, USA; ^2^Laboratory of Proteomics, Institute of Biology, Eötvös Loránd UniversityBudapest, Hungary

**Keywords:** information integration, consciousness, cortical state, anesthesia, ascending arousal system, neuronal state repertoire

## Abstract

States of consciousness have been associated with information integration in the brain as modulated by anesthesia and the ascending arousal system. The present study was designed to test the hypothesis that electrical stimulation of the oral part of the pontine reticular nucleus (PnO) can augment information integration in the cerebral cortex of anesthetized rats. Extracellular unit activity and local field potentials were recorded in freely moving animals from parietal association (PtA) and secondary visual (V2) cortices via chronically implanted microwire arrays at three levels of anesthesia produced by desflurane: 3.5, 4.5, and 6.0% (where 4.5% corresponds to that critical for the loss of consciousness). Information integration was characterized by *integration* (multiinformation) and *interaction entropy*, estimated from the statistical distribution of coincident spike patterns. PnO stimulation elicited electrocortical activation as indicated by the reductions in δ- and θ-band powers at the intermediate level of anesthesia. PnO stimulation augmented integration from 1.13 ± 0.03 to 6.12 ± 1.98 × 10^3^ bits and interaction entropy from 0.44 ± 0.11 to 2.18 ± 0.72 × 10^3^ bits; these changes were most consistent in the PtA at all desflurane concentrations. Stimulation of the retina with discrete light flashes after PnO stimulation elicited an additional 166 ± 25 and 92 ± 12% increase in interaction entropy in V2 during light and intermediate levels. The results suggest that the PnO may modulate spontaneous ongoing and sensory stimulus-related cortical information integration under anesthesia.

## INTRODUCTION

Information integration is fundamental to the proper functioning of the cerebral cortex in the wakeful conscious subject (Tononi, 2004). It has been proposed that two components are associated with the presence of consciousness: an immense repertoire of causal brain states (representing information), and the ability to functionally integrate this information (integration; Tononi et al., 1998; Alkire et al., 2008). Information integration in the brain is presumed to be disrupted in diminished states of consciousness such as anesthesia (Alkire, 1998; Schrouff et al., 2011), sleep (Tononi and Massimini, 2008), absence seizures (Blumenfeld and Taylor, 2003), vegetative state (Zhou et al., 2011; Boly et al., 2012), and coma (Noirhomme et al., 2010). Conversely, a restoration of information integration may be a requisite for the return of consciousness (Rosanova et al., 2012).

One way to examine information integration in neuronal networks is through multichannel extracellular recording of unit activity (UA) *in vivo*. Such recordings provide useful data on the distribution of extracellular spike firing rates as well as detailed spatiotemporal patterns of spike configurations relevant to neural information coding (Berry et al., 1997; Grun et al., 2002; Uzzell and Chichilnisky, 2004; Lawhern et al., 2011). In particular, coincident spike firing patterns (temporally synchronized firing) between neuronal populations may represent information flow (Mainen and Sejnowski, 1995) that leads to sensory processing and behavioral manipulations (Grun et al., 1999, 2002). Information processing that enables sensory perception, language generation, memory encoding and retrieval, and presumably conscious awareness, therefore, is influenced by the synchronization between neuronal circuits and networks (Singer and Gray, 1995; Engel et al., 1999a,b; Gray, 1999).

Cortical state, traditionally viewed as a function of the wake-sleep cycle, is under the precise control of the ascending arousal system (AAS; Lee and Dan, 2012) with origins in brainstem nuclei and extensive projections to the thalamus, basal forebrain, hypothalamus, and neocortex via ventral and dorsal pathways (Jones and Yang, 1985; Holstege and Kuypers, 1987). These states fall on a continuum that is heavily influenced and determined by fluctuations in spontaneous neuronal activity (Harris and Thiele, 2011). A major source of ascending projections from the AAS is the oral part of the pontine reticular nucleus (PnO; Moruzzi and Magoun, 1949; Jones and Yang, 1985; Jones, 2003). Microinjection of neostigmine or carbachol into the pontine reticular formation elicits an enhancement of rapid-eye-movement-sleep-like state in mouse and rat models, respectively (Bourgin et al., 1995; Lydic et al., 2002). Moreover, PnO efferent activity may be reduced during anesthesia (Sukhotinsky et al., 2006).

If cortical information processing is under the control of the AAS, and anesthesia suppresses AAS activity (Mashour et al., 2005; Brown et al., 2011; Lee and Dan, 2012), then a plausible question is whether exogenous activation of the AAS, in particular that of the PnO, during anesthesia may augment cortical information integration and presumably, shift the cortical state toward waking consciousness. To-date, no study has been performed to examine whether electrical stimulation of the PnO can increase information integration during continued anesthetic administration. Here we test the hypothesis that electrical stimulation of the PnO increases information integration in cortical neuronal networks of the rat *in vivo* at various depths of desflurane anesthesia from light sedation to a near surgical level. In order to gain information about higher order sensory integrative processing, multisite extracellular UA was recorded simultaneously from the secondary visual area (V2) and the adjacent parietal association cortex (PtA). To examine the effect of PnO stimulation on sensory integration, both spontaneous and visual evoked UA was tested on separate days. To characterize the depth of anesthesia and its effect on cortical state, local field potentials (LFP) were measured simultaneously with UA using the same electrode array. As we show, PnO electrical stimulation had a significant modulatory effect on cortical information integration under anesthesia.

## MATERIALS AND METHODS

### ANIMALS

All experimental procedures and protocols were approved by the Institutional Animal Care and Use Committee of the Medical College of Wisconsin (Milwaukee, Wisconsin). All procedures conformed to the Guiding Principles in the Care and Use of Animals of the American Physiologic Society and were in accordance with the Guide for the Care and Use of Laboratory Animals (National Academy Press, Washington, DC, USA, 1996).

Experiments were performed on eight adult (250–360 g), male, Sprague-Dawley rats (Harlan Laboratories, Madison, WI, USA). All animals were housed in a reverse light-dark cycle room for at least 10 days prior to surgical implantation, and remained there until all experiment protocols were completed. Food and water access was *ad libitum*.

### SURGICAL PREPARATION

Aseptic technique was used during surgical preparation. Animals were anesthetized, through spontaneous breathing, with 1.9 ± 0.2% isoflurane, vaporized into a mixture of 30% O_2_, 70% N_2_ and delivered at a flow rate of 5 L/min. Anesthesia was distributed through a gas anesthesia mask (Model 929-B Rat Gas Anesthesia Head Holder, David Kopf Instruments, Tujunga, CA, USA). Anesthetic concentration was monitored (POET IQ2 monitor; Criticare Systems, Inc., Waukesha, WI, USA) through a sampling line connected to the anesthesia mask. Core body temperature was rectally monitored (model 73A, YSI, Yellow Springs, OH, USA) and maintained at 37°C with a thermostat-controlled, electric (TC-1000, CWE Inc., Ardmore, PA, USA) heating pad.

To prepare the animal for surgery, Betadine (VWR, Radnor, PA, USA) and alcohol were repeatedly applied to the dorsal surface of the head. Sterile, 0.5% bupivacaine was administered subcutaneously to provide local anesthesia. A midline incision was made; the skin and connective tissue were reflected laterally to reveal the cranium. Hydrogen peroxide (in some cases a cautery) was used to stop any bleeding.

A low-speed, compressed air driven dental drill (DENTSPLY Professional, Des Plaines, IL, USA) was used to create a craniotomy (2 mm × 4 mm) above the PtA and V2 for the recording microelectrode array; the dura mater was resected to allow for penetration of the electrode array. For extracellular UA and LFP recording, a multi-shank 16-contact microwire array (wire diameter = 33 μm, electrode spacing = 500 μm, row separation = 1000 μm, tip angle = 45°; Tucker-Davis Technologies, Alachua, FL, USA) was implanted such that one row resided in V2 (4.8 mm posterior, +2.5 mm lateral, and -1.5 mm ventral from bregma) and the other row in PtA (3.8 mm posterior, +2.5 mm lateral, and -1.5 mm ventral from bregma). The reference wire, attached to the electrode array, was wrapped around a stainless-steel epidural screw (1 mm posterior, -3 mm lateral from bregma). Gel foam and silicone gel were applied around the periphery of the electrode array (Kwik-Sil, World Precision Instruments, Sarasota, FL, USA).

For brainstem stimulation, a concentric bipolar electrode (SNEX-100, David Kopf Instruments, Tujunga, CA, USA) was implanted into the PnO (8 mm posterior, -1.3 mm lateral, ventral = 8.2, and insertion angle = 15°) through a cranial burr hole. For visual stimulation, an 8-mm diameter light emitting diode (LED; Szabo-Salfay et al., 2001; peak wavelength = 660 nm, American Bright Optoelectronics Corp, Chino, CA, USA) was secured to the cranium posterior to the contralateral eye (approximately 4–5 mm anterior to bregma). At the chosen wavelength, light penetrates through the scalp, bone, and brain tissue (Maarek et al., 1984) and stimulates the retina directly, bypassing the optics of the eye. This obviates the need for eye lubrication and ensures the constancy of retinal illumination independent of the animal’s posture. Transcranial flash stimulation with a red LED has been shown to produce standard visual evoked potentials in rodents (Szabo-Salfay et al., 2001).

**FIGURE 1:**
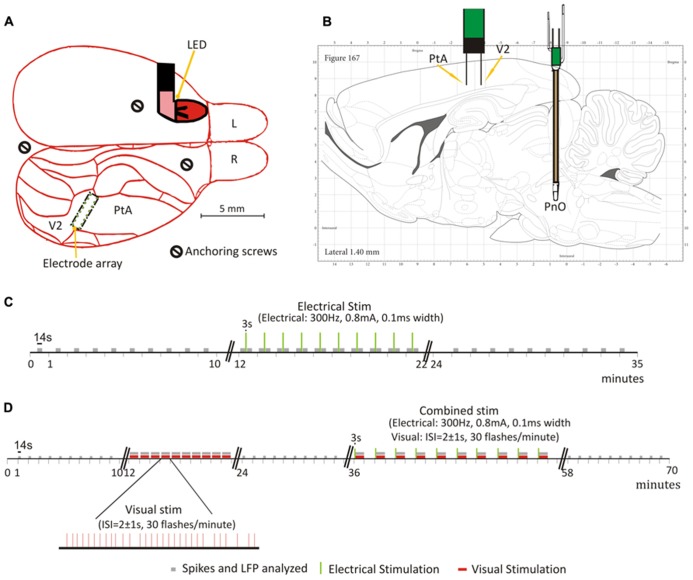
**Schematic of electrode placement and timelines of PnO and light flash stimulation experiments. (A)** Placement of the 16-wire electrode array in the rat secondary visual area (V2) and parietal association area (PtA), and light emitting diode (LED) behind the left eye. Schematic is overlaid on a dorsal view of the rat brain. Each green dot represents the location of a micro-wire. **(B)** Schematic overlaid, with permission, on a sagittal view of the rat brain from the Paxinos (Paxinos and Watson, 2007) rat brain atlas. The electrode array and concentric bipolar electrode, implanted in the oral part of the pontine reticular nucleus (PnO) for electrical stimulation, are illustrated. **(C)** Testing on day 1. Illustrated here is the progression of the experiment once steady state was reached at one desflurane concentration. This was repeated for all desflurane concentrations. Unit activity (UA) and local field potentials (LFPs) were recorded for the duration of the experiment. Electrical stimulation commenced 10 min after steady-state was reached at each desflurane concentration. **(D)** Testing on day 2. As with day 1, steady-state desflurane was reached at each concentration prior to testing. Light flashes, alone, were delivered 10 min after reaching steady-state desflurane. This was followed by paired stimulation, light flashes presented immediately after PnO stimulation, 10 min later. The desflurane concentration order was randomized on both days of testing. Green vertical lines represent the electrical stimulation. Gray bars represent the segments of data that were used in the analyses. Red bars represent the light flashes.

Additional anchoring screws were implanted immediately anterior to the interaural line and posterior to the LED. A schematic of the locations of the recording and stimulating electrodes, as well as the LED is displayed in **Figures 1A,B**. The assembly was secured in place with a gentamicin-enriched bone cement (Palacos R&G, Zimmer Orthopaedic Surgical Products, Dover, OH, USA) and cerebond skull adhesive (Leica Microsystems, Bannockburn, IL, USA). The analgesic carprofen (5 mg/kg subcutaneously once daily) and the antibiotic enrofloxacin (10 mg/kg subcutaneously once daily) were administered post-operatively for 2 and 7 days, respectively. Animals were housed individually to reduce the chance of inadvertent removal of the skullcap.

### EXPERIMENTAL PROTOCOL

Testing commenced no earlier than 7–10 days post-operatively. On the experimental day, rats were placed into a custom-built, transparent, plexiglass anesthesia experimental box (46 cm × 23.5 cm × 23 cm). Rats retained the ability to breathe spontaneously throughout the experiment. Desflurane and O_2_ (30%) were delivered at a flow rate of 5 L/min and carefully monitored (POET IQ2; Criticare Systems, Inc., Waukesha, WI, USA), and rat body temperature was controlled at 37°C throughout the duration of the experiment. Prior to testing, the rats were allowed 45 min to accommodate to a darkened room. The microelectrode array was then connected to a preamplifier via a headstage (Blackrock Microsystems, Salt Lake City, UT, USA) outside the anesthesia chamber. LFPs, UA, and time markers from both the visual and electrical stimuli were recorded using a 128-channel neural acquisition system (Blackrock Microsystems, Salt Lake City, UT, USA). LFPs were analog bandpass-filtered at 1–250 Hz, notch-filtered at 60 Hz, and digitally sampled at 1 kHz. Extracellular UA was auto-thresholded using a root mean square multiplier of -6.25. They were analog bandpass filtered from 250 to 7500 Hz, and digitally sampled at 30 kHz. Visual stimuli (30 flashes/min, 5 ms duration) were computer generated and delivered randomly with an interstimulus interval of 2 ± 1 s. LED flashes were generated by square-wave pulses slightly exceeding the LED saturation current (56 mA) so the luminance of the flash was always the same (1200 mcd/mm^2^). PnO stimulation parameters were guided by previous work (Antognini et al., 2003), and refined to fit our experimental design. Pilot data obtained from six rats suggested that a current intensity of 0.8 mA produced EEG desynchronization in the absence of gross behavioral movement (data not shown). PnO stimulation consisted of a 3 s train of 0.1 ms pulses delivered at 300 Hz at a current intensity of 0.8 mA using a constant current generator (Rys-Williams, Medical College of Wisconsin).

Each rat was tested under three desflurane concentrations: 3.5, 4.5, and 6%. These concentrations were chosen because loss of consciousness (LOC) is believed to occur within this range. The righting reflex, a surrogate measure of consciousness in rats (Franks, 2008), was barely present at 3.5% and was immediately lost at 4.5%. At 6% it was presumed that rats were unconscious. The order of concentrations was randomized for each rat on both testing days.

Summarized in **Figures 1C,D** are the protocols used for testing on day 1 (PnO stimulation alone) and day 2 (light flashes and PnO stimulation), respectively. Rats were allowed to equilibrate at each desflurane concentration for 20-min prior to testing and data acquisition. All eight rats were tested at 3.5 and 4.5% desflurane. Two animals were euthanized due to removal of the skullcap and, therefore, six rats were tested at 6% desflurane.

### DAY 1 – PnO STIMULATION ALONE

After the equilibration period at each desflurane concentration, spontaneous UA and LFP were acquired for 10 min. This was followed by PnO electrical stimulation: electric current was delivered once (3 s-on, 57 s-off) every minute for 10 min. Finally, 10 more minutes of spontaneous UA and LFP were recorded after PnO stimulation ceased. This process was repeated once at each desflurane concentration.

### DAY 2 – COMBINED STIMULATION (LIGHT FLASHES PRESENTED AFTER PnO STIMULATION)

To investigate how PnO stimulation would modulate the neuronal response to a visual stimulus, and subsequently the information integration of the system, a combined stimulation paradigm was used. Spontaneous UA and LFP, as in day 1, were acquired for 10 min after the initial equilibration period. This was followed by the presentation of the visual stimulus alone: light flashes (~5 ms duration) were delivered randomly, with an interstimulus interval of 2 ± 1 s, at a rate of 30/min throughout a 10-min recording session. Spontaneous UA and LFP were recorded for another 10 min. This was followed by the combined stimulation: light flashes (same parameters as above) were delivered immediately after PnO stimulation (same parameters used on Day 1): one cycle = 30 light flashes after each PnO stimulation. This was repeated for 10 cycles. Finally, 10 min of spontaneous UA and LFP were acquired after stimulation. This process was repeated once at each desflurane concentration.

### HISTOLOGICAL VERIFICATION

At the end of experimental testing, electrode placement was confirmed histologically on chemically fixed coronal sections. The rats were cardio-perfused with 300 ml 0.9% saline, followed by 250 ml 4% paraformaldehyde solution, through the heart ventricle. The brains were harvested and stored in the paraformaldehyde solution for 24 h, and subsequently transferred to 0.01 M phosphate buffered saline (pH 7.4). The 80 μm-thick coronal brain sections were cut by a vibratome (Vibratome Series 3000 Plus, Ted Pella, Inc., Redding, CA, USA). Brain slices were stained with cresyl violet Nissl and imaged, using a Nikon Eclipse E600 (Nikon Inc, Melville, NY, USA) microscope, to visualize the location of the electrode sites.

## DATA ANALYSES AND STATISTICS

The LFPs were used to gauge the state of anesthesia and the effectiveness of the PnO stimulation. Specifically, band powers (δ = 1–4 Hz, θ = 5–7 Hz, α = 8–12 Hz, β = 13–30 Hz, low-gamma *L* - γ = 30–50 Hz, and high-gamma *H* - γ = 70–140 Hz) were obtained from the spectra by averaging signal power in the respective frequency ranges.

### SPIKE SORTING

**FIGURE 2:**
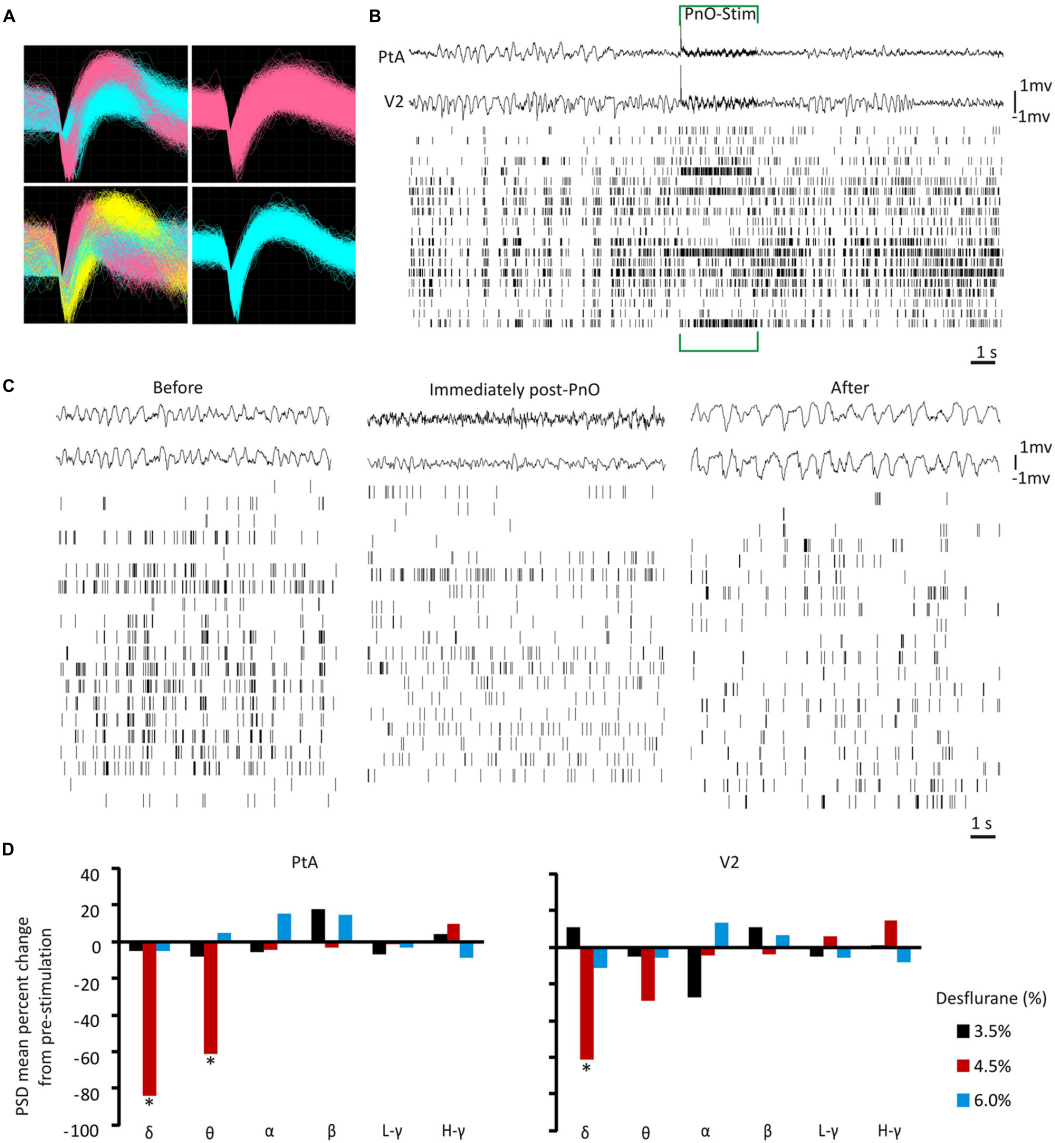
**Local field potentials and unit activity before and after PnO electrical stimulation. (A)** Representative waveforms of extracellular discharge recorded from 4 electrode wires in one animal. Each quadrant is from one electrode wire. A single electrode wire was able to record from multiple neurons. Colored waveforms represent distinct units after offline spike sorting. **(B)** Representative spike trains and local field potentials (LFPs) before, during and after PnO electrical stimulation at 4.5% desflurane in one rat. An induced artifact was present during the 3-s stimulation ON period, and analyses were carried out on data devoid of this artifact. **(C)** Example LFP and unit activity (UA) from one representative rat at 4.5% desflurane before and after PnO stimulation. At 4.5% desflurane the LFP was characterized by mostly high-amplitude activity, but became more desynchronized after PnO stimulation. The two LFP traces were chosen from each region (PtA = top waveform, V2 = bottom waveform). The spike raster plots show all active units from this rat. **(D)** LFP band power percent change from pre-stimulation. PnO stimulation elicited cortical arousal, as determined by the induced significant reductions in delta and theta band powers in the PtA at 4.5% desflurane. Delta band power was also reduced in V2 after PnO stimulation at 4.5% desflurane. No change in band powers was observed at 6.0% desflurane. *p < 0.05 for post- vs. pre-PnO stimulation. δ = 1–4 Hz, θ = 5–7 Hz, α = 8–12 Hz, β = 13–30 Hz, low-gamma *L* - γ = 30–50 Hz, and high-gamma *H* - γ = 70–140 Hz. PSD = power spectral density.

At each concentration, and for each recording from both testing days, the spike waveforms were sorted offline with PowerNAP (OSTG, Inc., Fremont, CA, USA) into individual neuronal units using principal component analysis. Cluster boundaries of discrete units were determined by *K*-means clustering. Remaining outliers were manually removed. Any movement and stimuli related artifacts were easily identified (synchronized across all recorded channels) and removed. An example of sorted spike waveforms is displayed in **Figure 2A**. The electrical stimulation elicited an artifact on both LFPs and UA; a representative spike train from one rat at 4.5% desflurane is displayed in **Figure 2B** to illustrate this artifact.

### DATA SEGMENTATION

To further prepare the data for subsequent analyses, pre-stimulus and post-stimulus components were classified for both the electrical and visual stimuli by using the timestamps of each respective stimulus as the reference time-series. More specifically, data were segmented as follows: pre-PnO stim (-14 to 0 s), post-PnO stim (3–17 s), pre-Flash (-500 to 0 ms), post-Flash (0–500 ms). Similar time-points were chosen to segment the spontaneous recordings. All segments were then concatenated for each of the spontaneous, pre-stimulus and post-stimulus recordings (yielding a total of 140 s of data/condition). As a result, one spike train was generated at each condition for every active unit. The data acquired during the electrical stimulation (3 s) was not used because of the presence of an induced artifact (see **Figure 2B**).

### VISUAL EVOKED RESPONSES

Sorted spike waveforms from both regions were used to characterize neuronal responses to the visual stimulation. Specifically, the conditional probability of a spike relative to the visual stimulus was determined using perievent histograms from -0.5 to 1 s (relative to stimulus onset), with a bin size of 10 ms, for each individual unit using NeuroExplorer (Version 4.091, Nex Technologies, Madison, AL, USA). In order to delineate responsive from unresponsive units, the mean firing rate and 95% confidence intervals were used. Units crossing the confidence intervals were considered responsive. In order to create an average perievent histogram from populations of neurons in V2 and PtA, population vectors were calculated. These vectors are weighted linear combinations of the histograms from each neuron.****The time to peak response was calculated from V2 responsive units. The amplitude of the visual evoked responses was normalized to the maximum value in each rat in each condition in order to more clearly see temporal changes.

### SPIKE PATTERNS

The number of distinct (unique) spike patterns observed over a given period of time was considered an approximate measure of a subset of the repertoire of local brain states. For this calculation, the original concatenated time-series was transformed into a binarized one (1 ms bin size); a value of 1 was assigned to each bin where a spike occurred, and a value of 0 when no spike occurred. The number of times each pattern appeared, across all active units, in the time series was normalized to the total number of patterns in the system. The resultant distribution was then used to calculate, via information-theoretic measures, the informational content within each cortical region (PtA and V2).

### INFORMATION INTEGRATION

Two closely related quantities were used to measure instantaneous information integration for coincidently firing units. Integration (Tononi et al., 1998), also known as total correlation or multi-information (Watanabe, 1960) is defined as:

$$I\left(X\right)=\overset{N}{\underset{i=1}{\sum }}H\left(x_{i}\right)-H\left(X\right)$$

where *x*_i_ is the activity of an individual unit, *H* (*x*_i_) is the entropy of the firing probability of each unit, and *H*(*X*) is the joint entropy probability of coincident spike patterns of all units in the system. Integration as defined above quantifies the average information shared among the units of a system. If the system is composed of units, which are statistically independent, then *I*(*X*) = 0. *I*(*X*) is maximum when all spikes are synchronized.

Information integration was also quantified as interaction entropy (Shew et al., 2011).

$$E\left(X\right)=H(X_{s})-H\left(X\right)$$

where *X*_s_ is a set of coincident firing patterns obtained from the randomized (shuffled) version of the measured spike trains. The uncorrelated spike firing events were removed by shuffling the data. The shuffling by a random time lag****of spikes decreases the spike correlations while the average spike rate of each unit is preserved. *E*(*X*) quantifies the portion of entropy accounted for by the spike correlations. Thus, *I*(*X*) and *E*(*X*) provide two different approximations of an information-based measure of unit interactions. Interaction entropy quantifies the amount of redundancy, owing to correlation within the neuronal population, in a system. As with *I*(*X*), *E*(*X*) = 0 if the system consists of statistically independent units.

### DYNAMIC CORRELATION

Coordination in unit activity was also characterized by calculating a more conventional measure, dynamic correlation (Beggs and Timme, 2012):

$$C_{xy}=\langle (x-\langle x\rangle )\left(y-\langle y\rangle \right)\rangle$$

where *x* and *y* are the binary activities of single units at a given time, 〈*x*〉 and 〈*y*〉 are the time-averaged firings of these two individual units. The fluctuation in firing of two individual units from their average at a particular time, is thus represented by the terms (*x* - 〈*x*〉) and (*y *- 〈*y*〉). Multi-channel spike correlation was computed by subtracting the mean of each channel, creating a correlation matrix, zeroing the diagonal, and computing the all pair-wise dynamic correlation for neurons in each brain region. This was done for each rat at all conditions from day 2 of testing and then averaged.

### STATISTICS

Data from day 1 and 2 were analyzed separately. The effect of PnO stimulation alone on LFP band powers was first tested with repeated measures ANOVA, with condition (pre-PnO, post-PnO), concentration, and region as independent variables, rat as the subject variable, and band powers (δ, θ, α, β, *L *- γ, H - γ) as response variables. This was then followed by examining the effect of PnO stimulation (day 1) on information integration with repeated measures ANOVA, with the condition (baseline, pre-PnO, post-PnO and recovery), concentration, and region as independent variables, rat as the subject variable, and number of unique patterns, the number of spikes, integration, and interaction entropy as dependent variables.

The effect of PnO stimulation on the visual stimulus-related information integration (day 2) was tested with repeated measures ANOVA with condition (baseline, recovery after PnO, recovery after Flash w/PnO, pre-Flash w/out PnO, post-Flash w/out PnO, pre-PnO, post-PnO, pre-Flash w/PnO, post-Flash w/PnO), concentration, and region as independent variables, rat as the subject variable, and the number of unique patterns, the number of spikes, integration, and interaction entropy as dependent variables.

The effect of PnO stimulation of the visual evoked response was tested with repeated measures ANOVA with condition (Flash, Flash w/PnO), and concentration as independent variables, rat as the subject variable and time to peak response as the dependent variable. Responsive neurons with post-flash firing exceeding the 95% confidence interval only were included in this test.

The effect of PnO stimulation on dynamic spike correlation was tested with condition (pre-PnO, post-PnO, pre-Flash, post-Flash, pre-Flash w/PnO, and post-Flash w/PnO), concentration, and region as independent variables, rat as the subject variable and dynamic correlation as the dependent variable.

All data were analyzed using custom scripts in MATLAB version 7.3.0 (MathWorks Inc., Natick, MA, USA). Statistical analyses were performed using NCSS 2007 (NCSS, Kaysville, UT, USA). All inferential statistics were performed on raw data. The data were tested for normality using the Shapiro–Wilk test, which yielded no reason to reject the normality assumption. For all data analyzed via repeated measures ANOVA, the sphericity assumption was determined; the Geisser–Greenhouse adjustment was made if the sphericity assumption was violated. All analyses were two tailed and a *p* < 0.05 served as the criterion for statistical significance. Statistical results are reported as either main effects or interaction from repeated measures ANOVA unless otherwise specified. All data are presented as ± standard deviation from the mean.

## RESULTS

### CORTICAL STATE AT DIFFERENT ANESTHETIC LEVELS

At 3.5% desflurane, the LFP was characterized by relatively low-amplitude, high-frequency (desynchronized) activity. As the desflurane concentration was increased, slower waves predominated at 4.5 and 6.0%. Power spectral analysis of the spontaneous LFPs revealed that δ power was highest at 4.5% [*F*(2,31) = 3.52, *p* = 0.042, and *p* < 0.05, T–K test] β power was reduced at desflurane concentrations exceeding 3.5% in both cortical regions [*F*(2,31) = 17.72, *p* < 0.0001, and *p* < 0.05, T–K test). None of the other band powers were affected by desflurane concentration, or region.

### EFFECT OF PnO STIMULATION ON CORTICAL STATE AND INFORMATION INTEGRATION

PnO stimulation modified the LFP such that a desynchronized pattern was observed in both PtA and V2 at the intermediate desflurane concentration of 4.5% (**Figure 2C**). This typically persisted for 14 s. The δ- and θ-band powers were reduced [*F*(2,69) = 8.28, *p* = 0.0006 and *F*(2,69) = 3.17, *p* = 0.048, respectively] at 4.5% desflurane in both regions (NS at 3.5 and 6%). *Post hoc* testing showed that δ-band power was suppressed by 84 ± 3 and 62 ± 9% in the PtA and V2, respectively (*p* < 0.05, T–K test). However, only θ-band power was reduced, from 0.006 ± 0.002 to 0.0025 ± 0.0021 μv^2^/Hz, in PtA (*p* < 0.05, T–K test) as illustrated in **Figure 2D**. None of the other band powers were affected by PnO stimulation.

PnO stimulation produced no change in the average spike rate [*F*(7,328) = 0.44, *p* = 0.88], or the number of active units [*F*(7,328) = 0.15, *p* = 0.99] at any desflurane concentration, suggesting that PnO stimulation influenced cortical synchrony, but not the overall level of neuronal activity. In contrast, PnO produced a substantial increase in the number of unique spike patterns in PtA (67 ± 8.5 to 86 ± 11.5) and in V2 (65 ± 14.7 to 82 ± 7), indicative of a rise in the repertoire of local brain states.

**FIGURE 3:**
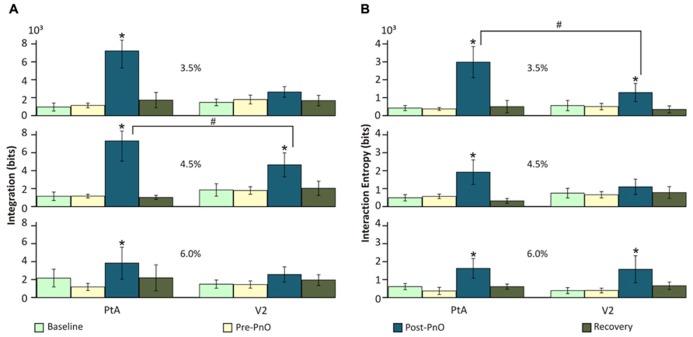
**Effect of PnO stimulation on integration and interaction entropy of cortical spike populations on day 1 PnO electrical stimulation produced robust increases in (A) integration and **(B)** interaction entropy in both PtA and V2.** These changes were generally larger and more consistent across the anesthetic levels in PtA than in V2. Integration was augmented significantly more in PtA than in V2 at 4.5% desflurane. *p < 0.05 post-PnO vs. pre-PnO. #*p* < 0.05 PtA vs. V2. Error bars are ±1 SD.

The analysis of UA from data obtained on day 1 revealed a significant overall effect of PnO stimulation on integration *I*(*X*). ANOVA revealed a significant interaction between desflurane concentration and PnO stimulation [*F*(6,145) = 3.24, *p* = 0.005] and between cortical region and PnO stimulation [*F*(3,145) = 3.73, *p* < 0.012). The effect of PnO stimulation was generally larger in PtA than in V2 (*p* < 0.05, T–K test). As displayed in **Figure 3A**, PnO stimulation increased overall integration, from 1.13 ± 0.03 to 6.12 ± 1.98 × 10^3^ bits, in PtA at all desflurane concentrations. In V2, a significant increase was evident at 4.5% desflurane only (*p* < 0.05, T–K test). Integration after PnO stimulation was 58 ± 4% higher in PtA than in V2 at 4.5% desflurane (*p* < 0.05, Student *t*-test).

Interaction entropy *E*(*X*) was also significantly altered by PnO stimulation. The effect depended on both desflurane concentration and brain region [*F*(6,145) = 2.27, *p* = 0.04 ANOVA interaction]. As displayed in **Figure 3B**, PnO stimulation increased overall interaction entropy, from 0.44 ± 0.11 to 2.18 ± 0.72 × 10^3^ bits, at all desflurane concentrations in PtA, and at 3.5 and 6.0% in V2 (*p* < 0.05, T–K test). Interaction entropy after PnO stimulation was 57 ± 6% smaller in V2 than in PtA at 3.5% desflurane (*p* < 0.05, Student *t*-test).

### FLASH-EVOKED RESPONSE BEFORE AND AFTER PnO STIMULATION

**FIGURE 4:**
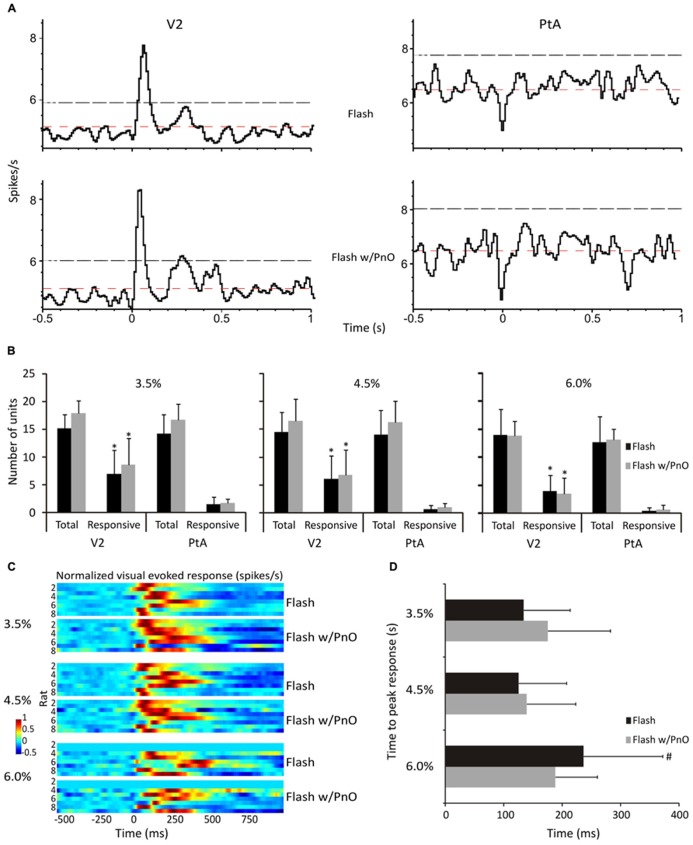
**Effect of desflurane and PnO stimulation on visual evoked responses. (A)** Population vectors of all neurons from both regions in one representative rat at 4.5% desflurane are displayed. Neurons in V2 responded in a stereotypic manner to light flashes, whereas PtA neurons remained unresponsive. Neurons were considered responsive if they crossed the 95% confidence intervals (black dashed lines). Also displayed, red dashed lines, are the expected mean firing rates. **(B)** The number of total recorded and responding neurons are displayed. In general, more V2 neurons responded to light flashes than those in PtA at all desflurane concentrations. **(C)** Visual evoked responses are displayed from responding V2 neurons in each rat to light flashes alone and paired with PnO stimulation. **(D)** The time to peak response was not altered by PnO stimulation. However, it took longer for V2 neurons to reach peak response at 6.0% desflurane. *p < 0.05 responding neurons in V2 vs. responding neurons in PtA. #*p* < 0.05 time to peak response at 6 vs. 4.5%. Error bars are ±1 SD.

Light flashes, presented to the retina, elicited stereotypic neuronal responses in V2 at all desflurane concentrations. Regional population vector responses to light flashes at 4.5% desflurane are illustrated in **Figure 4A**. The number of responding units was dependent on an interaction between brain region and desflurane concentration [*F*(2,69) = 3.36, *p* = 0.04]. As shown in **Figure 4B**, V2 units (5.7 ± 1.5) were more responsive than PtA units (0.91 ± 0.52) to light flashes at all desflurane concentrations (*p* < 0.05, T–K test). The time to peak response in responding V2 units to light flashes alone was dependent on desflurane concentration [*F*(2,31) = 3.31, *p* = 0.049). Responsive units took longer to respond at the highest (236.6 ± 135.4 ms) than at the intermediate (125 ± 81.8 ms) desflurane concentration (*p* < 0.05, T–K test), and this is illustrated in **Figure 4D**. As seen in **Figures 4A–D**, PnO stimulation did not alter the unit response to light flashes.

### EFFECTS OF PnO STIMULATION ON FLASH-EVOKED INFORMATION INTEGRATION

**FIGURE 5:**
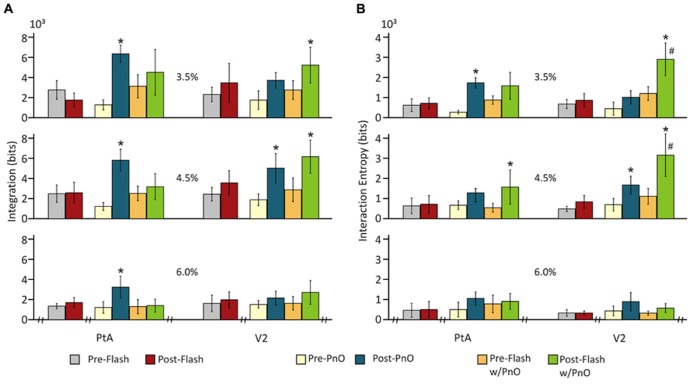
**Integration and interaction entropy before and after combined PnO and flash stimulation.** Visual stimulation (light flashes) alone did not affect **(A)** integration or **(B)** interaction entropy. PnO stimulation led, as in Day 1, to an increase in integration and interaction entropy. Light flashes presented after PnO stimulation elicited a large increase in integration and interaction entropy at 3.5 and 4.5% desflurane in V2. *post-stimulation vs. pre-stimulation. #post-Flash w/PnO vs. post-PnO. Significance: *p* < 0.05. Error bars are ±1 SD.

However, PnO stimulation greatly augmented the flash-induced increase in the number of unique patterns in V2 from 65 ± 11.6 to 128 ± 49.2 [*F*(7,328) = 2.74, *p* = 0.008 interaction, and *p* < 0.05, T–K test]. Consistent with this effect, PnO stimulation altered the integration values before and after flash stimulation (**Figure 5A**). There was a significant interaction between desflurane concentration and stimulation condition [*F*(16,332) = 2.26, *p* = 0.0039]. The presentation of light flashes alone failed to alter integration in either cortical region at any anesthetic concentration when compared with pre-Flash without PnO. Light flashes presented after PnO stimulation led to a 90 ± 16 and 114 ± 32% increase in integration at 3.5 and 4.5 % desflurane in V2 (*p* < 0.05, T–K test).

Interaction entropy values (**Figure 5B**) were dependent on the stimulus condition, desflurane concentration and cortical region [*F*(14,325) = 2.60, *p* = 0.001, ANOVA interaction]. As with integration, the presentation of light flashes alone failed to alter interaction entropy in either cortical region at any anesthetic concentration (*p* < 0.05, T–K test). However, light flashes presented after PnO stimulation elicited a 141 ± 17 and 183 ± 20% increase in interaction entropy at 3.5 and 4.5% desflurane, respectively in V2; an increase in interaction entropy was also observed at 4.5% in PtA.

To explore whether the presentation of light flashes after PnO stimulation could lead to a further increase in integration and interaction entropy, we compared Flash w/PnO vs. post-PnO. In general, light flashes presented after PnO stimulation were able to significantly increase interaction entropy by 166 ± 25 and 92 ± 12% in V2 at 3.5 and 4.5% desflurane, respectively [*F*(1,69) = 5.03, *p* = 0.048, and *p* < 0.05, T–K test].

**FIGURE 6:**
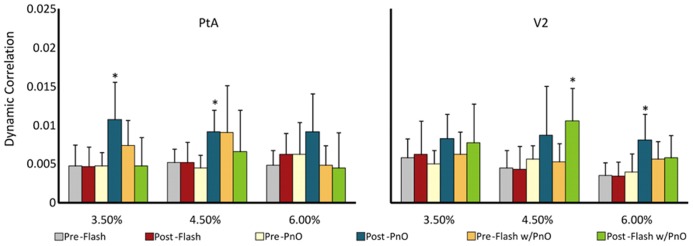
**Multi-channel dynamic spike correlation before and after combined PnO and flash stimulation.** PnO stimulation elicited large increases in dynamic spike correlation in PtA at the two lowest desflurane concentrations, and only at 6% in V2. Light flashes presented after PnO stimulation augmented dynamic spike correlation in V2 at the intermediate desflurane concentration. *p < 0.05 post vs. pre. Error bars are ±1 SD.

In order to characterize unit interactions with a more traditional measure, we also calculated the dynamic spike correlation across all units from testing on day 2. Dynamic spike correlation was examined before and after stimulation. Changes in the average of all pair-wise dynamic spike correlations were dependent on condition [*F*(5,107) = 8.08, *p* = 0.000002] and concentration [*F*(2,107) = 3.31, *p* = 0.04]. As illustrated in **Figure 6**, PnO stimulation led to an increase in dynamic correlation in the PtA at 3.5 (from 0.005 ± 0.002 to 0.011 ± 0.005) and 4.5% (from 0.004 ± 0.002 to 0.009 ± 0.003) desflurane, respectively (*p* < 0.05, T–K test) and at the highest concentration in V2 (*p* < 0.05, T–K test). Light flashes presented after PnO enhanced dynamic correlation in V2 at 4.5% desflurane from 0.005 ± 0.002 to 0.01 ± 0.004 (*p* < 0.05, T–K test).

## DISCUSSION

In this work****we examined the ability of PnO stimulation to modulate spontaneous and visual stimulus-evoked information integration in local cortical neuronal networks at three levels of anesthesia *in vivo*. The PnO was chosen as a target of stimulation because of its central role in the ascending modulation of cortical arousal (Lee and Dan, 2012). Emerging evidence suggests that the PnO may be one of the key targets in the neuromodulatory AAS as part of the mechanism for anesthesia induced unconsciousness (Devor and Zalkind, 2001; Lydic and Baghdoyan, 2005; Reiner et al., 2007; Sukhotinsky et al., 2007). To provide anesthesia, we used desflurane, a clinically used general anesthetic, due to its short equilibration time and ease of control at steady state. The desflurane concentrations used were near to that necessary to extinguish the righting reflex – a commonly used behavioral index of consciousness in rodents (Franks, 2008). Neuroelectrical recordings were performed in the parietal association and secondary visual cortices because of the putative roles of these regions in cortical information integration. We found that PnO stimulation produced electrocortical activation and simultaneously augmented information integration in both PtA and V2. In addition, PnO stimulation facilitated the light flash-evoked increases in information integration, especially in V2. Taken together, the data suggest that the PnO may play a role in the modulation of cortical state and integration of sensory information under a moderate depth of anesthesia.

### PnO AND CORTICAL STATE MODULATION

The PnO has been known to play a central role in the ascending modulation of cortical state, anesthesia and possibly even consciousness. It may exert comprehensive control over many arousal and sleep-wake promoting regions; the PnO is intimately linked to an array of subcortical and neocortical structures: cingulate cortices, hippocampus (HI), diagonal band of Broca, dentate gyrus, locus coeruleus (LC), mamillothalamic tract, preoptic area, medial septal nucleus, nucleus Basalis of Meynert, substantia nigra, subthalamic nucleus, and the cerebral cortex (Reinoso-Suarez et al., 2011). Its normal functioning depends heavily on glutamatergic and GABAergic cells that either facilitate or suppress cortical arousal and activation (Jones, 2003; Watson et al., 2008).

Previous studies have demonstrated that pharmacological activation of the PnO produces cortical activation in urethane-anesthetized animals (Fenik et al., 2005), decreases the propensity for sleep, and increases wakefulness (Tamminga et al., 1979; Camacho-Arroyo et al., 1991; Heiss et al., 2005; Watson et al., 2007). Furthermore, microinjection of muscimol, a GABA_A_ receptor agonist, into the pontine reticular formation of unanesthetized mice increases wakefulness and this effect can be blocked by administration of bicuculline (Flint et al., 2010). Our results reproduced the cortical arousal effect of pharmacological PnO activation using electrical stimulation. It is important to point out that neuronal activity was not recorded from the PnO or other components of the AAS, and we could not determine whether desflurane directly acted upon this region. In addition, the neurochemical cascade of events that transpired after stimulation of the PnO was not examined.

### EFFECT OF PnO STIMULATION ON INFORMATION INTEGRATION

A novel finding of this study was that PnO electrical stimulation had a significant modulatory effect on cortical information integration under anesthesia. Traditionally, information measures derived from spike trains are based on spike rates or interspike intervals. Multichannel data provide the next level of complexity, at which coincident spike firing patterns (temporally synchronized firing) between neuronal networks can be evaluated (Grun et al., 1999; Grun et al., 2002). Coordinated or coincident firing patterns are thought to represent information flow (Mainen and Sejnowski, 1995; Diesmann et al., 1999) that enables sensory perception, language generation, memory encoding and retrieval, and behavior. Moreover, conscious awareness is presumably influenced by the synchronization between neuronal elements and networks (Singer and Gray, 1995; Engel et al., 1999a,b; Gray, 1999).

In order to strengthen our confidence in the findings, in this work we used two measures of information integration, integration, and interaction entropy. Both of these measures were based on the statistical properties of coincident spike patterns. Expressed in units of bits, integration and interaction entropy quantify the average information shared among the units – hence information integration. The calculation of both quantities requires an estimation of the system’s entropy in a condition when its units are non-interacting. The latter is estimated by the sum of the entropies (integration) or by randomizing the spike timings (interaction entropy) of each unit, respectively. The first approach assumes that unit entropies would be the same if the units were truly independent and the second approach assumes that randomization completely removes the unit correlations. Obviously, with real data, none of these conditions are exactly satisfied, and integration and interaction entropy yield slightly different approximations. However, the results obtained with the two methods in this study were quite similar, reinforcing our confidence in the findings. These changes in entropy measures were confirmed by the increase in dynamic spike correlation after PnO stimulation. Finally, the measured, correlated firing may be due to direct interaction of the units or to common input, but this does not change the meaning of the calculated quantities.

A framework for further interpretation of our results is provided by the Integrated Information Theory of Consciousness (IITC; Tononi, 2010). Under this model, consciousness is an emergent property of brain complexity that scales with the brain’s ability to integrate information. Two necessary conditions for the latter are the large repertoire of distinct brain states (information) and the causal dynamics of these states or system elements (integration). It has been suggested that an essential component of anesthesia is a suppression of information integration (Alkire et al., 2008) and there has been some experimental support to this notion (Lee et al., 2009). It was therefore hypothesized that ascending cortical activation for example, by PnO stimulation, would work toward reversing the anesthetic effect on the state repertoire and information integration.

A possible measure of the local neuronal state repertoire is the number of unique spike patterns that occur over time. As predicted, the number of unique spike patterns increased after PnO stimulation, suggesting a transient increase in the information capacity. Quantified more directly, the observed increases in integration and interaction entropy after PnO stimulation could suggest, according to the IITC, a transient shift toward a conscious or REM-like state. Although we do not have objective knowledge of the state of mind of the rodent, we speculate that some form of conscious experience may be present in REM-like states. We consider REM sleep a form of conscious, although not wakeful, state as it can be accompanied by subjective experience during dreaming. A distinction between awareness and wakefulness is common in neurology (Laureys, 2005), and it has been suggested that consciousness may be akin to dreaming awake (Llinas and Pare, 1991). Even in the absence of overt behavioral expression, presumably blocked by the presence of the anesthetic in the spinal cord, such covert EEG changes have in fact been observed during nociceptive stimulation (Guignard et al., 2000; Avidan et al., 2008).

Of note is that increasing desflurane concentration from 3.5 to 6.0% did not diminish information processing. A limitation of our experiment design was that we did not study the awake condition, and therefore we were not able to tell if local information integration was already reduced at the 3.5% level compared to wakefulness. Perhaps it was, although it is also possible that neuronal interactions and local information integration in the anesthetized brain were relatively preserved (Ribeiro et al., 2010). Also, we do not know if electrocortical activity in PtA/V2 was altered by PnO stimulation to the same degree that it was in other brain regions, particularly in the frontal lobes that display substantial and characteristic changes in anesthesia (Cimenser et al., 2011). Thus, the reason for the apparent dissociation between frontal LFP and posterior information integration during increasing depth of anesthesia will have to be explored in future studies.

### EFFECT OF PnO STIMULATION ON VISUAL EVOKED RESPONSE AND INFORMATION INTEGRATION

In order to maximize the reproducibility of retinal stimulation, each rat was outfitted with a LED (Szabo-Salfay et al., 2001), emitting at a peak wavelength of 660 nm (allows for transillumination of the retina through skull and tissues), behind the left eye (contralateral to the recording microelectrode array and stimulating bipolar electrode; Maarek et al., 1984). As used before, this technique delivered consistent visual stimulation (discrete light flashes) without confounding changes in illumination due to postural adjustments of the animal and changes in optical properties of the eye. With this technique, visual evoked responses to the light flashes were readily elicited at all desflurane concentrations. Neurons in V2 were generally more responsive than those in PtA to light flashes both before and after PnO stimulation. The time to peak response was significantly increased at the highest desflurane concentration.

Although PnO stimulation altered neither the latency nor the magnitude of the visual evoked response, the information integration of the recorded units were greatly affected suggesting that PnO may modulate neuronal integration at a similar overall level of neuronal activity. PnO stimulation also augmented the dynamic spike correlation of unit activity during flash stimulation at the intermediate desflurane concentration. These findings provide further evidence that spike correlations may be an essential feature of neuronal encoding or decoding of stimulus-related information (Montani et al., 2009; Ohiorhenuan et al., 2010; Oizumi et al., 2010; Shimazaki et al., 2012).

Previous studies suggest that increased attention and arousal, due to manipulations of the AAS, dramatically improves encoding of a sensory stimulus (Zohary et al., 1994a,b; Bermudez Contreras et al., 2013), increases the neuronal responses to selective attention tasks (Reynolds and Chelazzi, 2004), and enhances the responses to a natural movie stimulus in the rat (Goard and Dan, 2009). Given the robust, flash-induced increases in unique spike patterns and information integration after PnO stimulation, we speculate that priming the activity of the PnO, together with other components of the AAS (Lee and Dan, 2012), may increase the readiness of the cortex to process and integrate incoming sensory information. Even though the direct effect of PnO stimulation decayed during flash presentation, its facilitating effect on the flash-induced increases in integration and interaction entropy were sustained in the post-stimulus period suggesting a relatively prolonged change in cortical state after PnO stimulation.

Taken together, our results support a role for the PnO in modulating cortical state and information integration during both spontaneous ongoing activity and visual stimulation. Future work could focus on pairing other sensory modalities to cortical state via manipulations of the AAS from multiple target sites, including the hypothalamus (Kelz et al., 2008) or basal forebrain (Pillay et al., 2011). These studies should contribute to a better understanding of the neuronal interaction of anesthetics and the AAS in modulating cortical information integration and the state of consciousness.

## Conflict of Interest Statement

The authors declare that the research was conducted in the absence of any commercial or financial relationships that could be construed as a potential conflict of interest.
